# Prevalence of metabolic syndrome among people living with human immunodeficiency virus in sub-Saharan Africa: a systematic review and meta-analysis

**DOI:** 10.1038/s41598-024-62497-y

**Published:** 2024-05-22

**Authors:** Yordanos Sisay Asgedom, Tsegaye Melaku Kebede, Amanuel Yosef Gebrekidan, Mengistu Meskele Koyira, Gedion Asnake Azeze, Afework Alemu Lombebo, Amelework Gonfa Efa, Kirubel Eshetu Haile, Gizachew Ambaw Kassie

**Affiliations:** 1 Department of Epidemiology, Wolaita Sodo University, Wolaita Sodo, Ethiopia; 2 Institute of Health, Jimma University, Oromia, Ethiopia; 3 School of Public Health, Wolaita Sodo University, Wolaita Sodo, Ethiopia; 4 School of Nursing and Midwifery, Hawassa University, Sidama, Ethiopia; 5 School of Medicine, Wolaita Sodo University, Wolaita Sodo, Ethiopia; 6 School of Nursing, Wolaita Sodo University, Wolaita Sodo, Ethiopia

**Keywords:** HIV, AIDS, Metabolic syndrome, Meta-analysis, Sub-Saharan Africa, Systematic review, Public health, Endocrine system and metabolic diseases

## Abstract

Metabolic syndrome (MetS) poses a significant clinical challenge for individuals living with HIV (PLHIV). In sub-Saharan Africa (SSA), this condition is becoming a growing concern, owing to lifestyle changes and an increasingly aging population. Several SSA countries have reported on the prevalence of MetS. However, these estimates may be outdated because numerous recent studies have updated MetS prevalence among PLHIV in these countries. Moreover, prior research has focused on various study designs to report the pooled prevalence, which is a methodological limitation. Therefore, this systematic review and meta-analysis aimed to determine the pooled estimates of MetS in PLHIV in SSA by addressing these gaps. We systematically searched Google Scholar, Science Direct, Scopus, Web of Sciences, EMBASE, and PubMed/Medline for the prevalence of MetS and its subcomponents among people with HIV in sub-Saharan Africa. The estimated pooled prevalence was presented using a forest plot. Egger’s and Begg’s rank regression tests were used to assess evidence of publication bias. Twenty-five studies fulfilled the inclusion criteria after review of the updated PRISMA guidelines. The pooled prevalence of MetS was 21.01% [95% CI: (16.50, 25.51)] and 23.42% [95% CI: (19.16, 27.08)] to the National Cholesterol Education Program Adult Treatment Panel III (NCEP/ATP III) and International Diabetes Federation (IDF) criteria, respectively. Low levels of high-density lipoprotein cholesterol (Low HDL) at 47.25% [95% CI: 34.17, 60.33)] were the highest reported individual subcomponent, followed by abdominal obesity at 38.44% [95% CI: (28.81, 48.88)]. The prevalence of MetS is high in sub-Saharan Africa. Low HDL levels and increased waist circumference/abdominal obesity were the most prevalent components of MetS. Therefore, early screening for MetS components and lifestyle modifications is required. Policymakers should develop strategies to prevent MetS before an epidemic occurs.

PROSPERO: CRD42023445294.

## Introduction

Human Immunodeficiency Virus (HIV) infection is a chronic, incurable disease that can lead to acquired immunodeficiency syndrome (AIDS) if untreated with antiretroviral therapy^1^. HIV remains a key public health burden, and globally 38.4 million people were living with HIV, and 650,000 deaths were reported by the World Health Organization (WHO) in 2021. More than two-thirds of people live with HIV worldwide, with nearly 25.6 million people living in Africa^2^. The increase in life expectancy and reduction in deaths are attributed to the success of highly active antiretroviral therapy (HAART) among HIV patients (PLHIV); however, the emergence of several cardiometabolic perturbations overshadows the decline in morbidity and mortality in HIV patients^3–5^. "cardio-metabolic perturbations” include a collection of concurrent metabolic risk factors, for instance, dyslipidemia, abdominal obesity, arterial hypertension and impaired glucose metabolism^6^.

Numerous complex mechanisms are yet to be clarified in the pathophysiology of MetS. MetS results from environmental, lifestyle, genetic, and epigenetic factors^7^. Most activated pathways of MetS are triggered by visceral adiposity, which is caused by high-calorie intake^8,9^. Insulin resistance, chronic inflammation, and neurohormonal activation are the proposed mechanisms for the progression of MetS^10^.

Metabolic syndrome (MetS) has long been used as an indicator of metabolic illness^11^. Metabolic syndrome (MetS) has become a growing concern in HIV-infected individuals over the last two decades^12^. Metabolic disorders in patients with HIV are caused by insulin resistance, aging, lifestyle changes, ART, and the virus itself. Lipid imbalances can occur during the natural course of an HIV infection.

People with MetS have sign and symptoms such as high blood pressure, high triglyceride, central obesity, and dyslipidemia and insulin resistance. People with insulin resistance may have acanthosis nigericans, which is darkened skin area on the back of neck, armpits and under the breast^13^.

MetS prevalence among PLHIV estimates ranging from 11.2 to 45.4%^14,15^. In SSA, HIV has been a pandemic for several years and remains a primary concern at the expense of similar dangerous diseases. While attention has been focused on the HIV pandemic, the emergence and spread of other equally dangerous health problems have remained unabated in several SSA nations. MetS exacerbates disease burden through clinical and biochemical links in HIV-positive patients. Several studies have reported a link between anti-retroviruses^5,16^. Studies from SSA have shown that the prevalence of MetS can reach up to 21.5%^17^. This difference may be due to differences in sample size, demographic characteristics, and criteria for the measurement of MetS^15^.

Many studies conducted in sub-Saharan African (SSA) countries have reported a prevalence of metabolic syndrome (MetS) of 21.5%^17^ however, there is a need to update this estimate, as numerous studies have been published since 2019 that provide new estimates on the prevalence of MetS among people living with HIV (PLHIV) in SSA countries. It is important to note that previous work did not exclusively focus on PLHIV and used various study designs (RCT, cohort, case–control, and cross-sectional) to report the pooled prevalence of MetS, which is a methodological limitation of their work. Therefore, this systematic review and meta-analysis aimed to determine the pooled estimates of MetS among PLHIV in SSA, using primary studies in the region.

This study offers evidence on the prevalence of MetS that can be crucial for policy makers for decision-making in healthcare regarding MetS prevention and patient management among PLHIV. It might be used by program directors to develop effective interventions to integrate care plans for PLHIV in the region and focus on risk reduction among PLHIV before the onset of MetS. Furthermore, pooling the prevalence of MetS among PLHIV will allow for a reduction in existing disparities and aid in the development of preventive and management strategies for healthcare services. As a result, this systematic review and meta-analysis was conducted to estimate the pooled prevalence of MetS among PLHIV in sub-Saharan Africa.

## Methods

### Reporting and protocol registration

This systematic review and meta-analysis were based on the recommended methodology and followed the Preferred Reporting Items for Systematic Review and Meta-Analysis for Protocols (PRISMA-P) 2020^18^ (Supplementary Table S1). The results were reported based on the PRISMA statement, and the article screening and selection process were demonstrated using a PRISMA-P flow diagram. The study was registered with the International Prospective Register of Systematic Reviews (PROSPERO) (registration number: CRD: 42023445294).

### Search strategy

We used different electronic biomedical databases and indexing services such as Google Scholar, Science Direct, Scopus, Web of Sciences, EMBASE, and PubMed/MEDLINE to search for relevant articles. Potentially applicable studies were manually searched for using a list of references from the retrieved studies. The search was limited to articles published in English. The search terms used for this systematic review and meta-analysis were 'prevalence,’ ‘Epidemiology,’ ‘magnitude,’ ‘burden,’ ‘metabolic syndrome,’ ‘metabolic disease,’ ‘metabolic disorder,’ ‘HIV/AIDS,’ ‘Africa’ and ‘Sub-Saharan Africa.’ Studies relevant to MetS prevalence were considered. The search strategy involved using keywords with “Medical Subjects Headings (MeSH)” and “All fields” by connecting “AND” and “OR” as necessary. The search was conducted between February 15th and March 12, 2023, by three authors (YSA, GAK, and MMK) who thoroughly examined various sources and databases following a rigorous methodology. The search strategy details are provided in a separate file (Supplementary Table S2).

### Eligibility criteria (inclusion and exclusion criteria)

The following criteria were used to include studies: (1) study type, both observational and experimental; (2) study period, studies published from database inception until December 2022; (3) study area, studies conducted in sub-Saharan Africa; (4) population, people living with HIV aged 18 years; and (5) Published in the English Language. Case reports, case series, review articles, and letters to editors, articles reported other than NCEP-ATP III and IDF criteria were excluded.

### Data extraction

Endnote citation manager for Windows Version X8 (Thomson Reuters, Philadelphia, PA, USA) was used to import the retrieved studies, and duplicates were removed. Four independent reviewers screened all the articles for the eligibility criteria. Reviewers began by screening the abstracts and titles, followed by full-text screening. Disagreements were resolved by inviting a fifth investigator to participate. Microsoft Excel with a standardized extraction format was used by two investigators for the data extraction. The Excel spreadsheet included the first author’s name, sample size, publication year, country, study design, impaired fasting glucose, elevated blood pressure, high triglyceride (TG), low level of high-density lipoprotein (low HDL), and prevalence of MetS according to the criteria of (National Cholesterol Education Program and Expert Panel on Detection, Evaluation, and Treatment of High Blood Cholesterol in adults (Adults Treatment Panel III)) and IDF (International Diabetes Federation). According to the PICO statement: Population: People living with HIV in SSA; Intervention: Exploring MetS; Comparison: Studies reporting MetS among people living with HIV outside SSA; Outcome: Proportion of MetS.

### Statistical analysis

STATA version 14.2 Statistical software (StataCorp, College Station, Texas, USA) was used for the analysis, and heterogeneity was checked across studies by computing the I^2^ statistical test. We assumed no, low, medium, and high heterogeneity across the studies if the I^2^ values were 0%, 25%, 50%, and 75%, respectively. A random effects model was used to analyze the pooled estimated prevalence with 95% confidence intervals (CI) using the “metaprop” command, since significant heterogeneity was detected between studies. Funnel plots for visual inspection and Egger’s and Begg’s rank tests were used to assess the evidence of publication bias. A forest plot was used to report the estimated pooled prevalence of MetS and its subcomponents.

### Outcome measurement

This study aimed to gather and analyze data from various studies conducted across sub-Saharan Africa to determine the pooled prevalence of MetS among people living with HIV in SSA, according to the NCEP ATP III and IDF criteria. The researchers used a systematic approach to identify relevant studies and extract relevant data. They then employed statistical methods to combine data from different studies and estimate the overall prevalence of MetS among people living with HIV in SSA.

These criteria are considered a subset of the following medical conditions or disorders:

1. Hypertension: systolic blood pressure > 130 mmHg or Diastolic blood pressure > 85 mmHg or pharmacologic Hypertension treatment
2. Abdominal obesity: Waist circumference of > 102 cm for men and > 88 cm for women
3. Dyslipidemia: Triglyceride (TG) 150 mg/dl or pharmacologic treatment
4. Hyperglycaemia: Fasting glucose > 100 mg/dl or pharmacologic treatment Dyslipidemia (Low HDL)
5. High-density lipoprotein cholesterol (HDL): < 40 mg/dl for men and < 50 mg/dl for women or pharmacologic treatment

In the case of NCEP-ATP III, three of the aforementioned criteria are utilized as diagnostic variables Similarly, in the IDF criteria, abdominal obesity (defined as waist circumferences of 94 cm for men and 80 cm for women) in combination with two of the aforementioned criteria listed above are considered^19,20^.

## Results

### Search results

**Figure 1:**
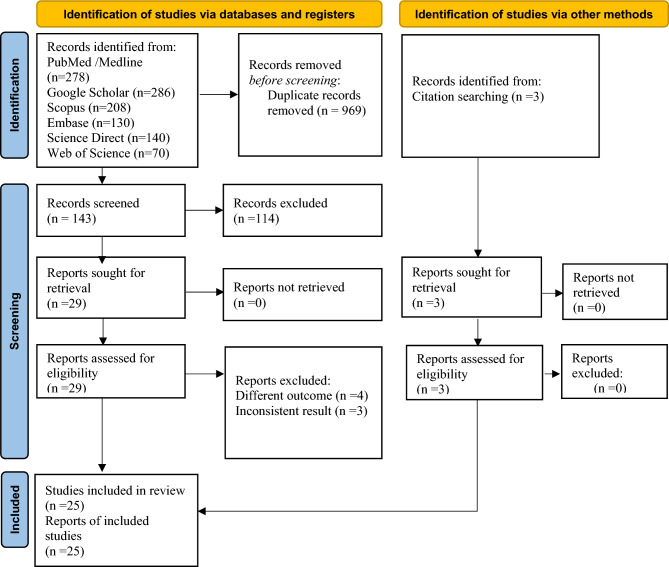
PRISMA flow diagram of the selection process of studies on MetS prevalence among PLHIV in sub-Saharan Africa in 2023.

A total of 1112 articles were initially identified using different biomedical databases, and 969 duplicates were excluded. Of the remaining 143 studies, 114 were excluded after reviewing their abstracts and titles. The full texts of the remaining 29 studies were downloaded and assessed to fulfil the required criteria. We again excluded seven studies (N = 4, different outcomes of Interest and N = 3, inconsistent results). Using citation search, we found three articles that met all inclusion criteria. Finally, 25 studies that met the inclusion criteria were included in this review using the search strategy, and duplicates were excluded using the endnote citation manager. Figure 1 illustrates the process of literature review, screening, and eligibility assessment of the study articles, and Supplementary Table S3 illustrates the details of the exclusion criteria.

### Characteristics of included studies

Among the 25 included studies, eight were from Ethiopia^21–28^, six were from South Africa^29–34^, three were from Nigeria^35–37^, two were from Kenya^38,39^, two were from Zambia^40,41^, and one each was from Cameroon^42^, Uganda^43^, Botswana^44^, and Zimbabwe^40^. The included study sample sizes ranged from 79^45^ to 1108^41^, with a total of 8602 people living with HIV/AIDS. Observational and experimental studies were conducted before July 2023, were conducted. The estimated pooled prevalence of MetS was assessed according to NCEP/ATP III and IDF criteria. Table 1 summarizes the baseline characteristics of the included studies. All the articles had a cross-sectional design and were facility-based. More than ten studies have been published since 2018. Nineteen studies defined MetS according to the NCEP/ATP III criteria, and 18 studies used the IDF criteria.

**Table 1:** Baseline characteristics of the included studies for the prevalence metabolic syndrome among people living with HIV in sub-Saharan Africa 2023.

Authors	Year		Country		Study design		Sample size	DM/impaired fasting glucose	Raised BP	High TG		Low HDL	Abdominal obesity	NCEP-ATP III	IDF
Berhane et al.^21^		2012	Ethiopia		CS		313	24.9	35.1	26.5		NA	13.7	21.1	NA
Tesfaye et al.^22^		2014	Ethiopia		CS		374	33.5	23.9	45.2		53.7	52.7	18	25
Hirigo et al.^23^		2016	Ethiopia		CS		185	51.3	9.7	NA		NA	NA	17.8	24.3
Bosho et al.^24^		2018	Ethiopia		CS		286	17.2	38.4	29.9		49.3	18.7	23.5	20.5
Bune et al.^28^		2019	Ethiopia		CS		422	28.4	56.9	37		34.3	28	22.5	NA
Ataro et al.^26^		2020	Ethiopia		CS		375	25.1	10.9	41.9		64.5	59.2	22.1	26.7
Gebrie et al.^27^		2020	Ethiopia		CS		415	NA	NA	NA		NA	41.3	NA	24.6
Bune et al.^25^		2020	Ethiopia		CS		438	56.2	55.6	37.1		34.3	40.8	NA	43.4
Fourie et al.^31^		2010	South Africa		CC		300	29.7	50	17.9		54.8	13.9	15.2	21.1
Muhammed et al. ^36^		2013	Nigeria		CS		200	3	9.5	16		68.5	NA	NA	21
Mbukneh et al.^42^		2013	Cameroon		CS		173	26.5	24.7	12.1		43	36.8	15.6	NA
Ayodele et al.^37^		2012	Nigeria		CS			291	18.6	28.2	13.1	54.6	19.2	12.7	17.2
Sobieszczyk et al.^29^		2016	South Africa		CS			160	1.3	NA	11.4	58.8	38.9	8.7	NA
Mashniya et al.^30^		2015	South Africa		CS			214	4.7	26.2	23.8	43.8	NA	9.6	NA
Guira et al.^47^		2016	Burkina Faso		CS			300	29.6	66.7	50	68.5	NA	NA	18
Muyanja et al.^43^		2016	Uganda		CS			250	NA	5.2	29.6	85.6	NA	58	NA
Tladi et al.^44^		2021	Botswana		CS			794	0.8	44.7	47.4	49.4	47.9	NA	26.8
Chihota et al.^40^		2022	Zambia and Zimbabwe		CS			420	12.2	42.4	10.2	63.4	41.9	21.9	18.5
Hamooya et al.^41^		2021	Zambia		CS			1108	17.6	30.6	13.1	25.3	27.9	26.4	NA
Masyuko et al.^38^		2018	Kenya		CS			300	18	2	30	17.9	55	NA	16.9
Ojong et al.^35^		2022	Nigeria		CS			150	45.3	29.3	38.6	90.6	66.6	32	50.3
Osoti et al.^39^		2020	Kenya		CS			300	3.5	22	10.5	26.1	18.8	6.3	NA
Phalane et al.^32^		2018	South Africa		CS			114	5.56	40.1	1.66	1.34	94.6	NA	28
Nguyen et al.^48^		2017	South Africa		CS			748	5	NA	1	1.3	11.7	24.1	26.5
Awotedu et al.^34^		2010	South Africa		CS			86	14	42.5	28.4	51.4	42.5	26.6	22.7

### Overall pooled prevalence estimates of MetS using ATP III and IDF criteria

**Figure 2:**
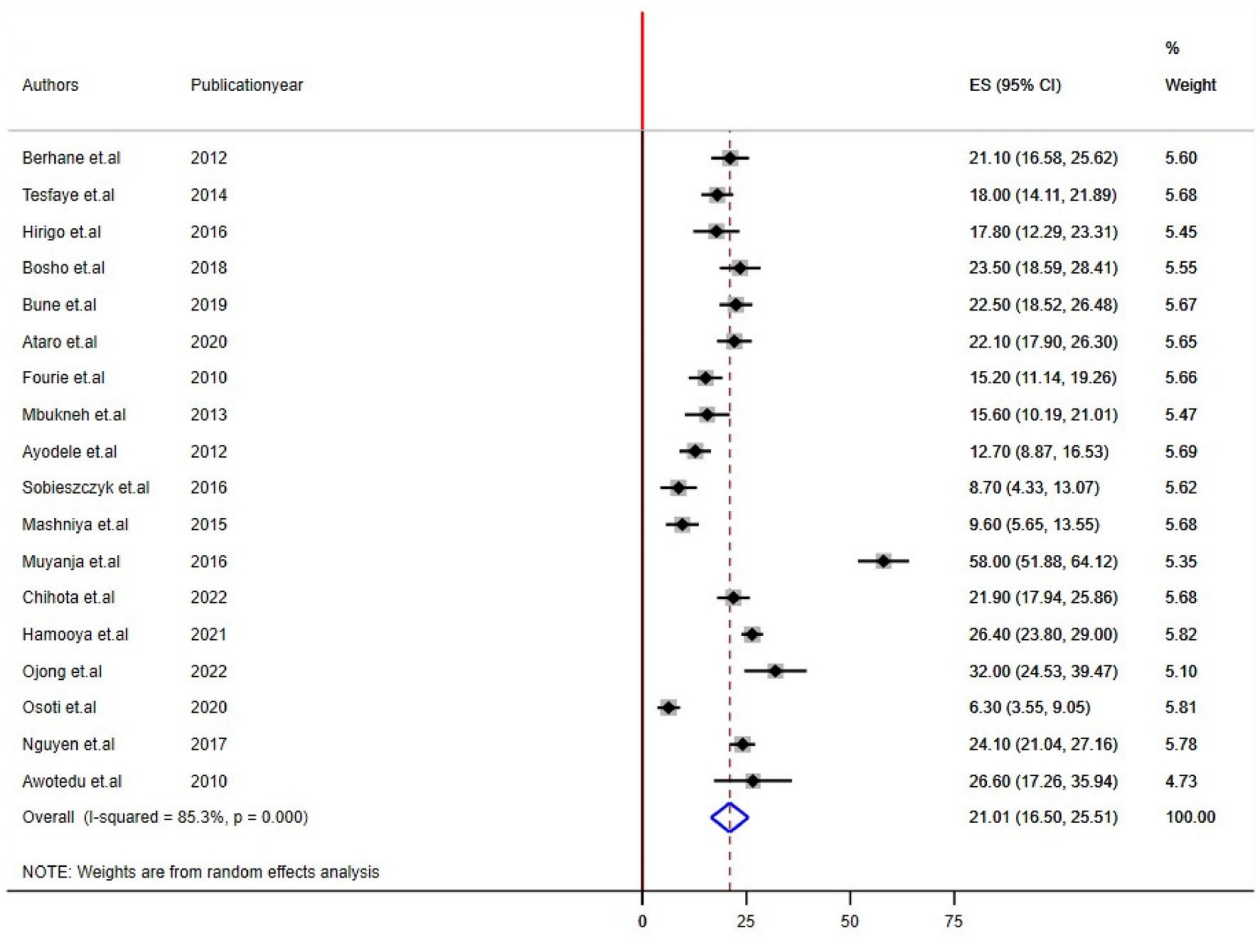
Forest plot depicting the overall pooled prevalence estimate of MetS among PLHIV in sub-Saharan Africa using NCEP/ATP III criteria.

**Figure 3:**
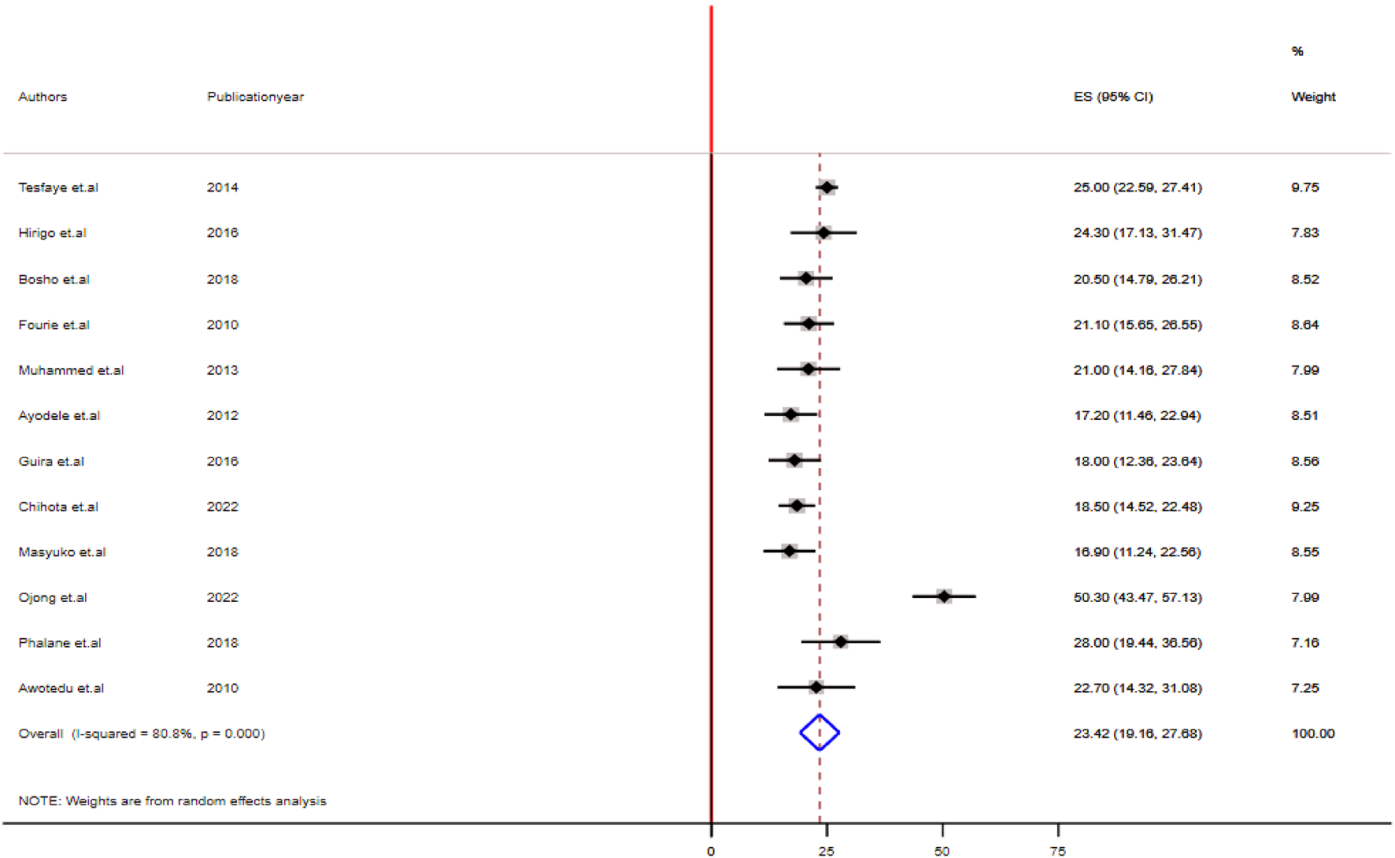
Forest plot depicting the overall pooled prevalence estimate of MetS among PLHIV in sub-Saharan Africa using the IDF criteria.

The estimated pooled prevalence of MetS among people living with HIV in SSA was 21.01% [(95% CI: 16.50, 25.51); I^2^ = 85.3%, P < 0.001] using NCEP/ATP III criteria (Fig. 2) and 23.42% [(95% CI: 19.16, 27.08); I^2^ = 80.8%, P < 0.001]) using IDF criteria (Fig. 3).

### Sub-components of metabolic syndrome

**Figure 4:**
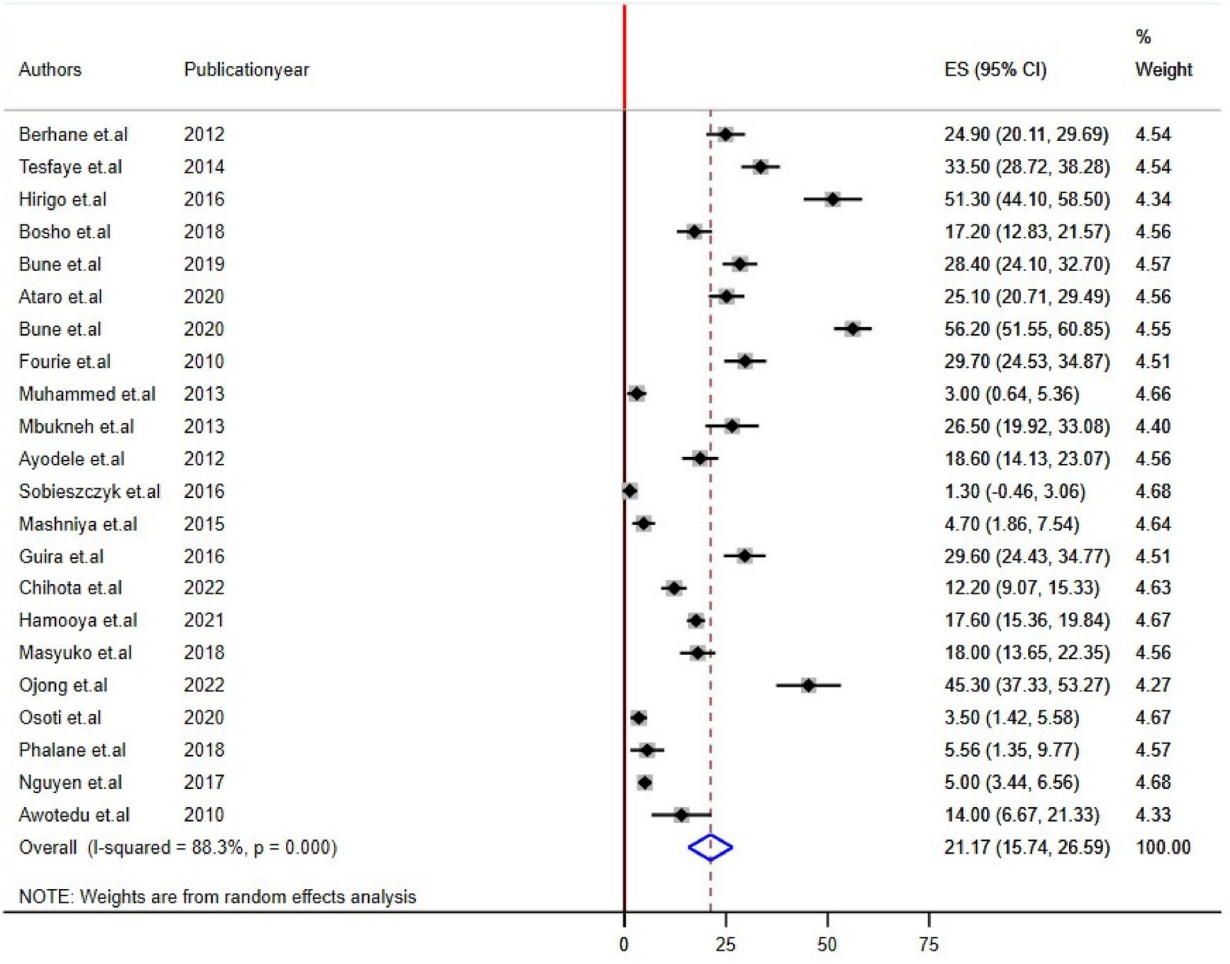
Forest plot depicting the overall pooled prevalence estimate of hyperglycemia among PLHIV in sub-Saharan Africa in 2023.

**Figure 5:**
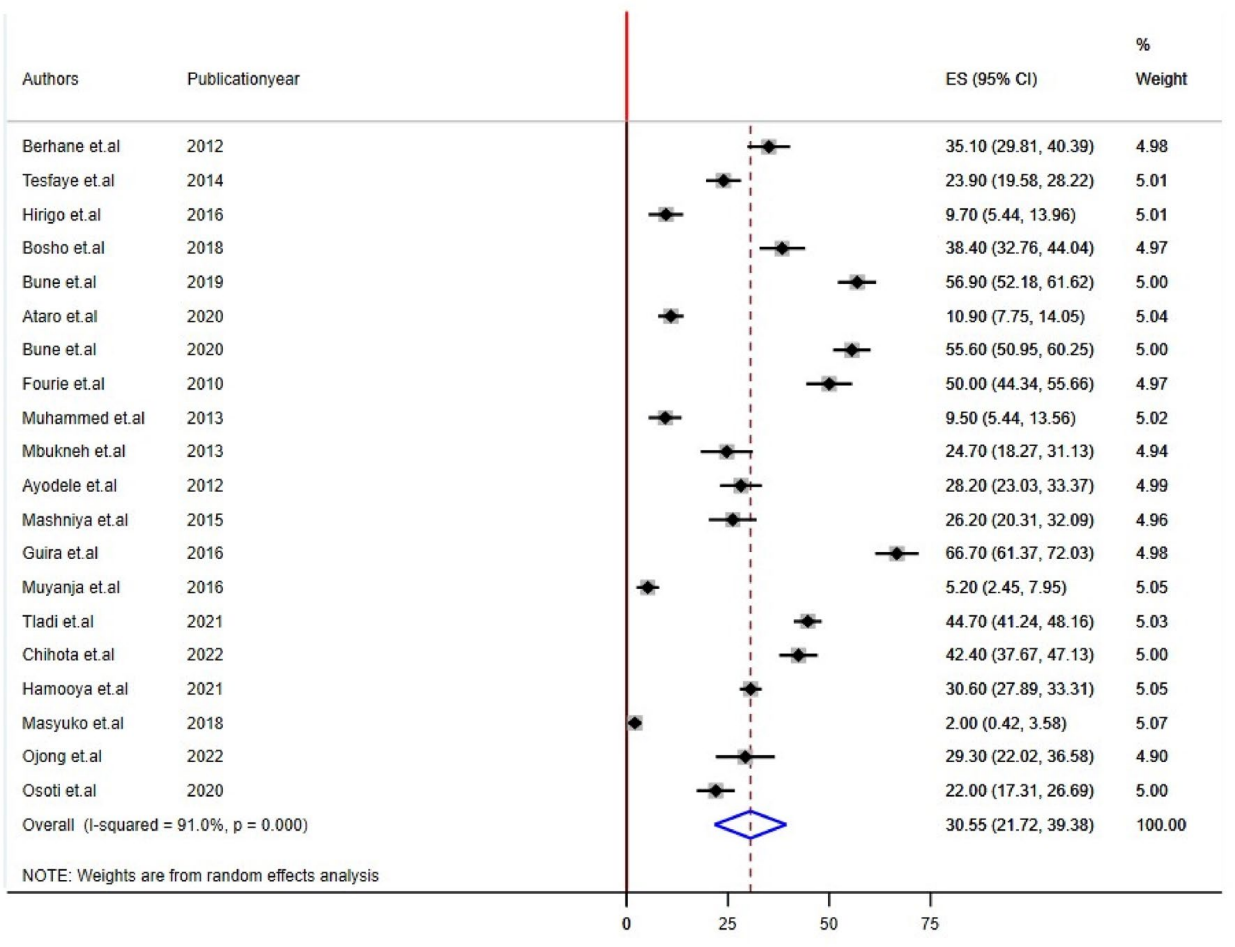
Forest plot depicting the overall pooled prevalence estimate of raised blood pressure among PLHIV in sub-Saharan Africa in 2023.

**Figure 6:**
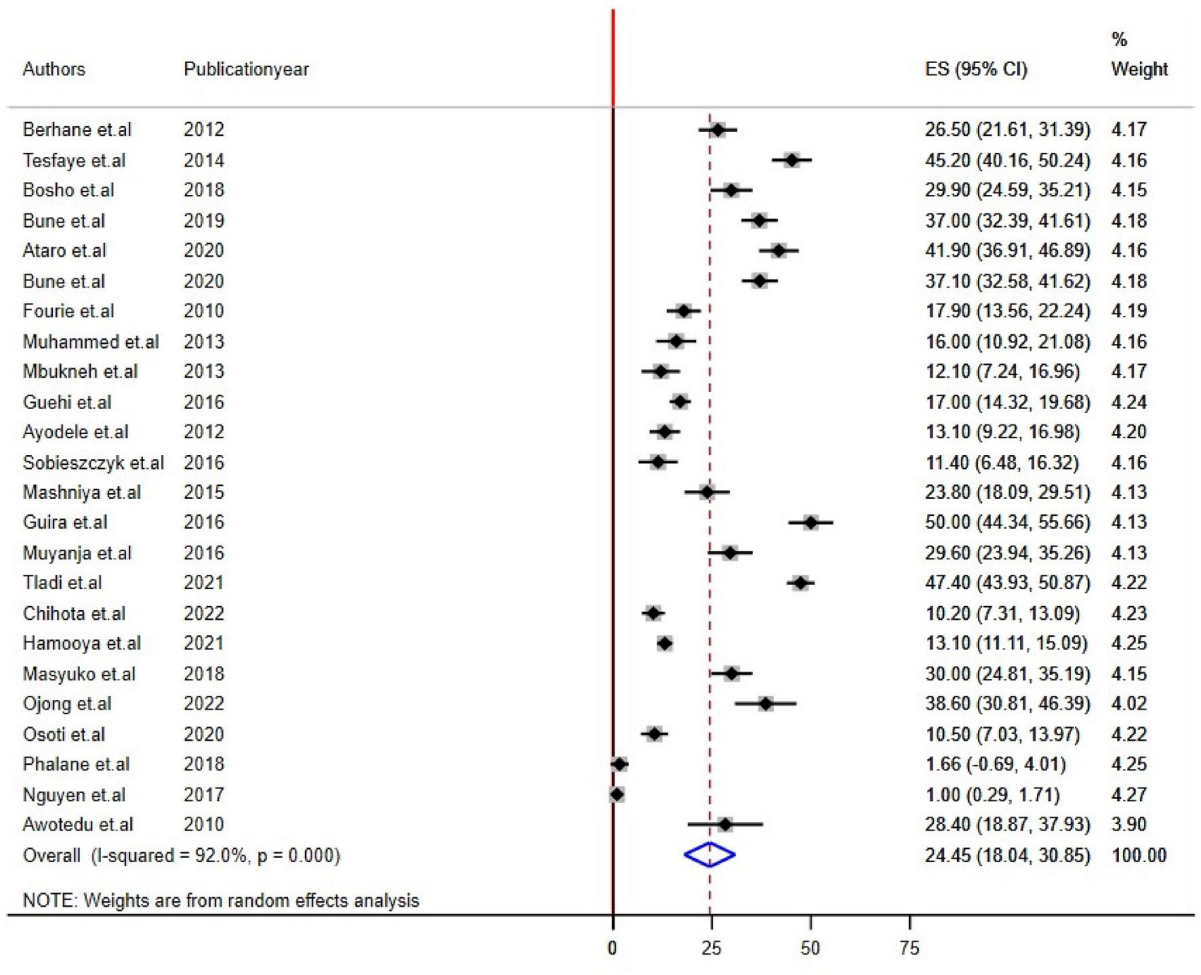
Forest plot depicting the overall pooled prevalence estimate of elevated triglyceride among PLHIV in sub-Saharan Africa in 2023.

**Figure 7:**
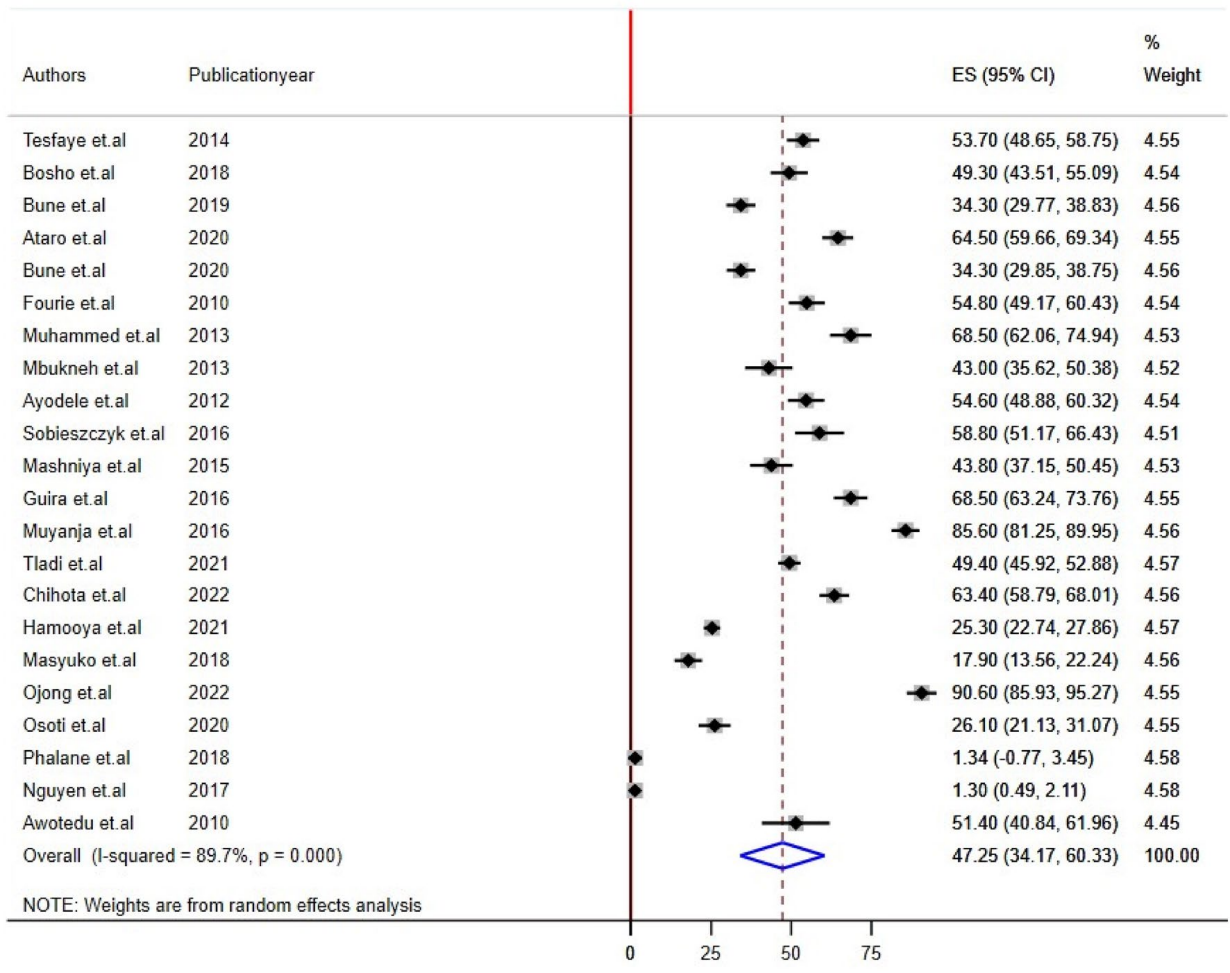
Forest plot depicting the overall pooled prevalence estimate of LDL-C among PLHIV in sub-Saharan Africa 2023.

**Figure 8:**
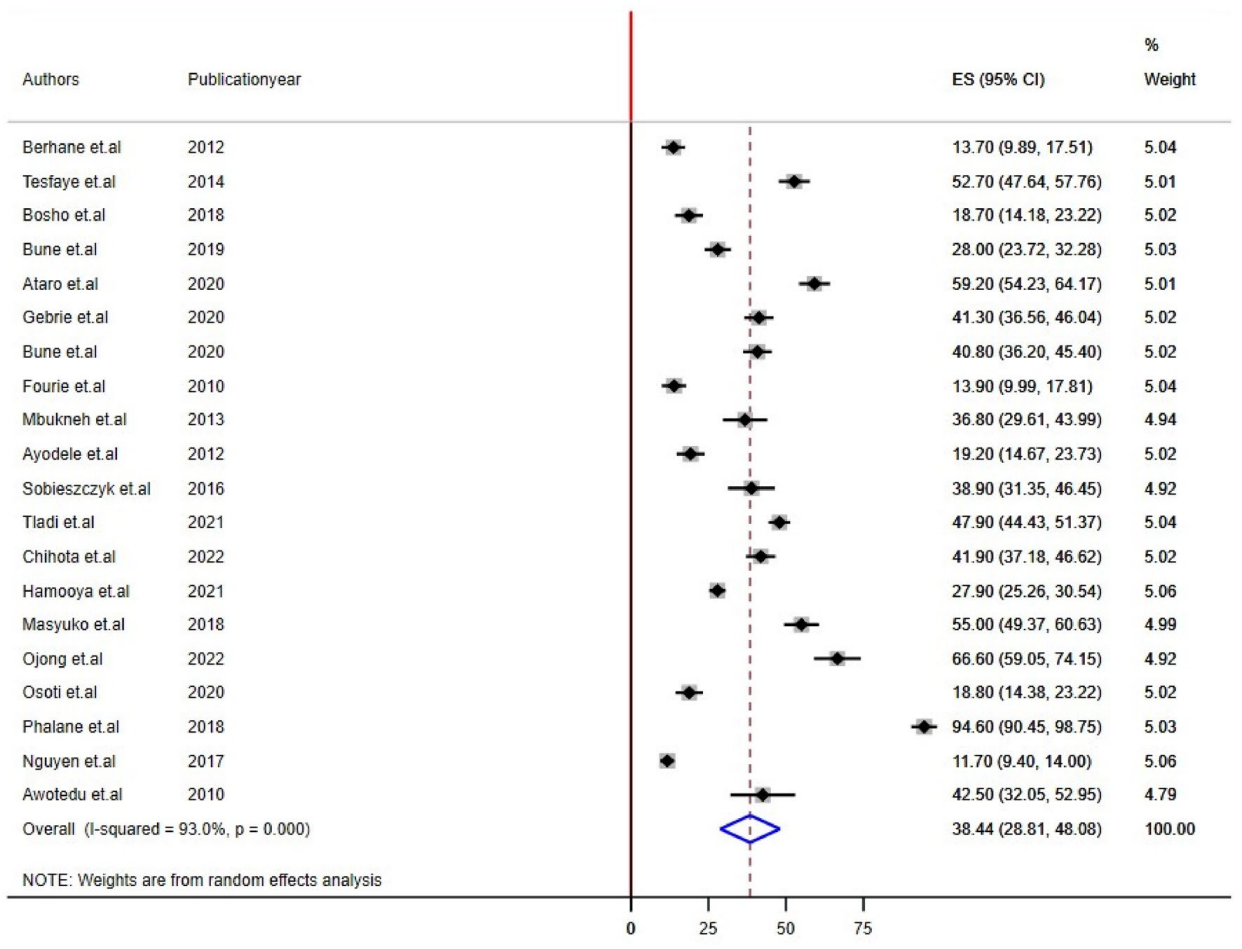
Forest plot depicting the overall pooled prevalence estimate of abdominal obesity among PLHIV in sub-Saharan Africa in 2023.

Twenty-two studies reported impaired fasting glucose and Low HDL levels^22,24,26,28–32,34–44,46–48^, 24 studies reported high triglyceride levels, and 20 studies reported hypertension/raised BP and increased waist circumference/abdominal obesity^21,22,24–29,31,32,34,35,37–42,44,45,48^ prevalence among PLHIV. The sub-components of MetS prevalence differed significantly among studies conducted in sub-Saharan Africa for PLHIV. The estimated prevalence of subcomponents was 21.17% [(95% CI: 15.74, 26.50), I^2^ = 88.3%, P < 0.001] for impaired fasting glucose, 30.55% [(95% CI: 21.72, 39.38), I^2^ = 91.0%, P < 0.001] for raised blood pressure, 24.55% [(95% CI: 18.04, 30.85), I^2^ = 92.0%, P < 0.001] for high triglyceride levels, 47.25% [(95% CI: 34.17, 60.33), I^2^ = 89.7%, P < 0.001] for low HDL, and 38.44% [(95% CI: 28.81, 48.88), I^2^ = 93.0%, P < 0.001] for abdominal obesity/increased waist circumference (Figs. 4, 5, 6, 7, 8).

### Publication bias

Funnel plots and Egger and Begg rank statistical tests at a 5% significance level were used to evaluate the presence of publication bias. The funnel plot showed symmetry (Supplementary Fig. S4) for pooled estimates using the NCEP-ATP III, and the Egger and Begg rank tests were not statistically significant (P-value = 0.258 and P-value = 0.225, respectively). Additionally, the funnel plot was almost symmetric (Supplementary Fig. S5) for IDF pooled estimates, and the Egger and Begg rank tests did not provide statistical evidence for the presence of publication bias (P-value = 0.863 and P-value = 0.158, respectively).

### Sensitivity analysis

**Figure 9:**
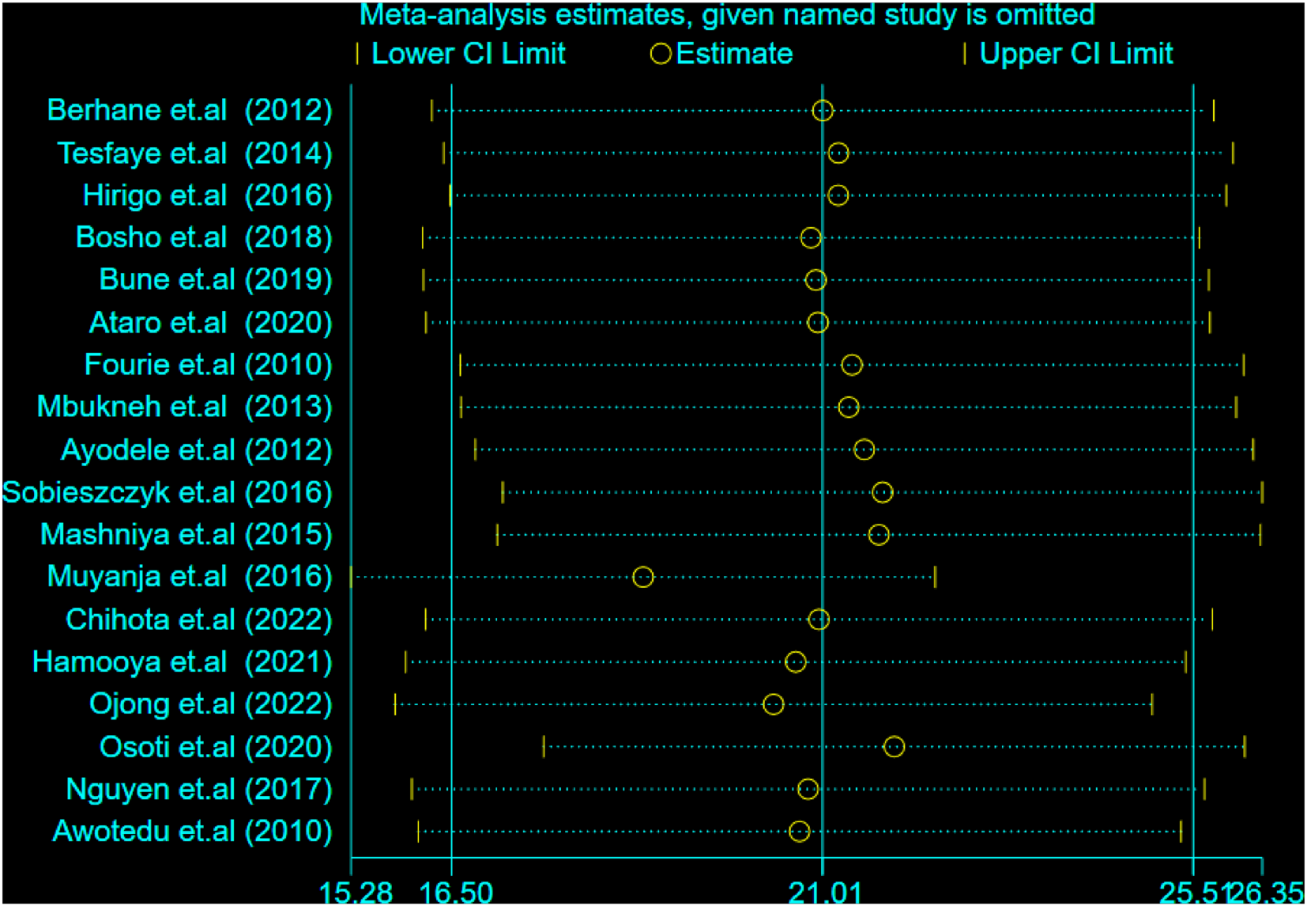
Sensitivity analysis for single study effect of estimated pooled prevalence based on NCEP/ATP III 2023.

**Figure 10:**
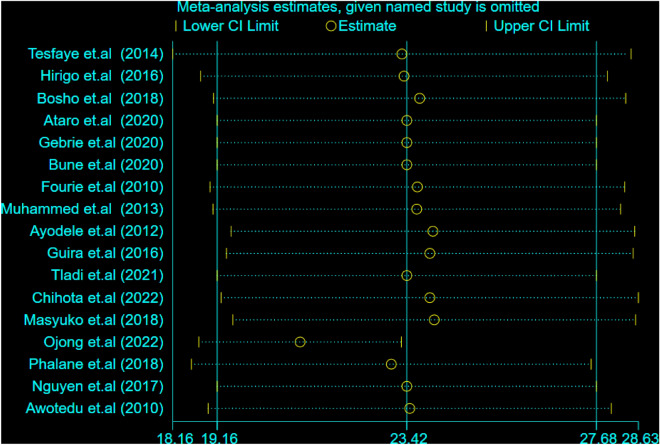
Sensitivity analysis for single study effect of estimated pooled prevalence based on IDF 2023.

By excluding each study individually, a leave-out-one sensitivity analysis was used to determine the effect of a single study on the pooled prevalence of MetS among people living with HIV in sub-Saharan Africa. According to our findings, no single study had a significant impact on the pooled prevalence of MetS among people living with HIV in sub-Saharan Africa using the NCEP-ATP III and IDF (Figs. 9 and 10).

## Discussion

The review found that among people living with HIV in sub-Saharan Africa, the prevalence of metabolic syndrome (MetS) was 21.01% according to NCEP/ATPIII criteria and 23.42% based on IDF criteria. Common components of MetS in this population included low HDL levels, increased waist circumference, and elevated blood pressure. This review is consistent with a similar review that found a high estimated pooled prevalence of MetS according to IDF criteria^17^. The use of a similar case definition for MetS might be attributed to the similarity of the studies. Likewise, the pooled prevalence in this study based on the NCEP/ATP III was comparable with reports from a global systematic review (20.6%)^49^ and sub-Saharan Africa (19.9%)^17^. This suggests that the findings of this study are consistent with those of previous research on MetS in PLHIV. These findings highlight the need for routine screening and management of MetS in PLHIV in SSA to reduce the risk of cardiovascular disease and other related complications. Further research is needed to understand the underlying mechanisms and risk factors associated with MetS and to inform targeted interventions to prevent and manage MetS in this vulnerable population.

In the present review, Low HDL (47%) was a common subcomponent of MetS, which is comparable to reports from Brazil^50^ and Italy^51^. Low HDL-C reflects an atherogenic dyslipidemia phenotype. Atherogenic dyslipidemia is a central lipoprotein associated with the development of MetS^52^. Atherogenic dyslipidemia can inhibit insulin metabolism through apoptosis and dysfunction of pancreatic beta cells^53–55^. Although the relationship between HDL function and MetS is not well established, it is likely that the relationship between HDL and insulin resistance also affects the development of MetS. Low HDL is mainly a result of apoA-I dysfunction (low levels of HDL in the blood) and systematic low-grade inflammation^56^. The second highest individual component of MetS was increased blood pressure/hypertension (30%). This finding was supported by studies from a systematic review and meta-analysis^57^. Antiretroviral therapy for PLHIV is commonly associated with hypertension (30%). The other MetS sub-component observed to be prevalent in this study was increased waist circumference (38%). This finding is consistent with that of a previous review^58^. The justification for the high prevalence of low HDL raised blood pressure/hypertension and increased waist circumference as individual components of MetS among PLHIV in sub-Saharan Africa might be due to the use of HAART. HAART has been shown to increase lipid levels and may not return to normal levels, leading to a high prevalence of low HDL levels. Additionally, the use of HAART in PLHIV is commonly associated with hypertension, resulting in a high prevalence of elevated blood hypertension. Finally, increased waist circumference and adiposity are also prevalent among HIV/AIDS patients and contribute to the high prevalence of MetS in this population. Therefore, appropriate interventions are necessary to prevent and manage MetS in PLHIV, including routine screening, lifestyle modifications, and pharmacological interventions, when necessary. Moreover, other MetS sub-components, such as high triglycerides (24%) and impaired fasting glucose (21%), are major among PLHIV in sub-Saharan, which is consistent with a previous review^58^. To mitigate the risk of metabolic syndromes, the latest HIV guidelines emphasize the importance of lifestyle modifications, including weight loss, increased physical activity, avoidance of substance abuse, and the implementation of medical interventions such as lipid-lowering therapy and antihypertensive drugs^59^.

Our review has clinical practice and public health implications; the clinical practice implications are healthcare providers should regularly evaluate HIV patients for metabolic syndrome components, including abdominal obesity, hypertension, elevated blood sugar levels, and abnormal lipid profiles. Early identification enables timely interventions and management. Individualized treatment plans tailored to the patients' specific risk factors and comorbidities are essential. These plans may include lifestyle modifications, medication adjustments, and regular follow-up visits. Collaborative care from professionals across different specialties, such as infectious disease specialists, endocrinologists, dieticians, and mental health professionals, is crucial for the comprehensive management of metabolic syndrome in HIV patients. Public health campaigns should raise awareness about the increased risk of metabolic syndrome in people living with HIV and promote healthy lifestyle behaviors to prevent and manage metabolic disturbances. Improving access to healthcare services for HIV patients, including the screening, diagnosis, and treatment of metabolic syndrome, is essential. This may involve reducing barriers to care, increasing healthcare provider training, and expanding the healthcare infrastructure. Continued research on the prevalence, risk factors, and outcomes of MetS in HIV populations is necessary to inform public health policies and interventions. Surveillance systems can help track trends and monitor the impact of interventions over time are the public health implications.

## Strength and limitation of the study

This review adhered to the PRISMA guidelines and conducted a thorough literature search across multiple databases to identify relevant studies. Although the meta-analytical methods applied in this study were robust, the findings must be interpreted with caution because of the limitations of the study. Heterogeneity was observed among the studies included in the meta-analysis. In addition, the study only included data from ten countries, which restricts the representativeness of the findings.

## Conclusion

The prevalence of MetS among PLHIV in sub-Saharan Africa is relatively high. Our review revealed that one in five study participants had MetS. Low high-density lipoprotein (HDL) levels, increased waist circumference, and increased BP pressure were more common in this review. We recommend early screening and appropriate interventions, including lifestyle modifications and pharmacological treatment, may be essential to prevent and manage MetS in PLHIV. Routine follow-up clinical and biochemical monitoring is also recommended. Additionally, creating awareness among PLHIV about MetS prevention, diagnosis, and treatment to prevent further complications is crucial. Promoting regular physical exercise, developing legislation for health promotion, and fighting obesity must be developed as a policy to address the modifiable risk factors of MetS among PLHIV. Furthermore, implementing novel interventions, such as integrated care plans for PLHIV in the region that can help strengthen the overburdened health system that also deals with other communicable diseases, can be an essential action.

### Supplementary Information

Supplementary Information.

## Data Availability

Data will be available based on the request of the corresponding author.

## References

[1] Blood GAC (2016). Human immunodeficiency virus (HIV). Transfus. Med. Hemother..

[2] World Health Organization (2021). The Global Health Observatory: Explore a World of Health Data.

[3] Grinsztejn B, Luz PM, Pacheco AG, Santos DV, Velasque L, Moreira RI (2013). Changing mortality profile among HIV-infected patients in Rio de Janeiro, Brazil: Shifting from AIDS to non-AIDS related conditions in the HAART era. PLoS One.

[4] Murray CJ, Ortblad KF, Guinovart C, Lim SS, Wolock TM, Roberts DA (2014). Global, regional, and national incidence and mortality for HIV, tuberculosis, and malaria during 1990–2013: A systematic analysis for the Global Burden of Disease Study 2013. Lancet.

[5] Palella FJ, Delaney KM, Moorman AC, Loveless MO, Fuhrer J, Satten GA (1998). Declining morbidity and mortality among patients with advanced human immunodeficiency virus infection. N. Engl. J. Med..

[6] Expert Panel on Detection, Evaluation, and Treatment of High Blood Cholesterol in Adults (2001). Executive summary of the third report of the National Cholesterol Education Program (NCEP) expert panel on detection, evaluation, and treatment of high blood cholesterol in adults (Adult Treatment Panel III). JAMA.

[7] Dizaji BF (2018). The investigations of genetic determinants of the metabolic syndrome. Diabetes Metab. Syndr. Clin. Res. Rev..

[8] Matsuzawa Y, Funahashi T, Nakamura T (2011). The concept of metabolic syndrome: Contribution of visceral fat accumulation and its molecular mechanism. J. Atheroscler. Thromb..

[9] Pekgor S, Duran C, Berberoglu U, Eryilmaz MA (2019). The role of visceral adiposity index levels in predicting the presence of metabolic syndrome and insulin resistance in overweight and obese patients. Metab. Syndr. Relat. Disord..

[10] Fahed G, Aoun L, Bou Zerdan M, Allam S, Bou Zerdan M, Bouferraa Y (2022). Metabolic syndrome: Updates on pathophysiology and management in 2021. Int. J. Mol. Sci..

[11] Rosolova H, Nussbaumerova B (2011). Cardio-metabolic risk prediction should be superior to cardiovascular risk assessment in primary prevention of cardiovascular diseases. EPMA J..

[12] Alvarez C, Salazar R, Galindez J, Rangel F, Castaãeda M, Lopardo G (2010). Metabolic syndrome in HIV-infected patients receiving antiretroviral therapy in Latin America. Braz. J. Infect. Dis..

[13] Medicine JH. Metabolic syndrome.

[14] Branson, B. M., Owen, S. M., Wesolowski, L. G., Bennett, B., Werner, B. G., Wroblewski, K. E. *et al*. Laboratory testing for the diagnosis of HIV infection: Updated recommendations (2014).

[15] Paula AA, Falcão MC, Pacheco AG (2013). Metabolic syndrome in HIV-infected individuals: Underlying mechanisms and epidemiological aspects. AIDS Res. Ther..

[16] Young F, Critchley JA, Johnstone LK, Unwin NC (2009). A review of co-morbidity between infectious and chronic disease in Sub Saharan Africa: TB and diabetes mellitus, HIV and metabolic syndrome, and the impact of globalization. Glob. Health.

[17] Todowede OO, Mianda SZ, Sartorius B (2019). Prevalence of metabolic syndrome among HIV-positive and HIV-negative populations in sub-Saharan Africa—A systematic review and meta-analysis. Syst. Rev..

[18] Page MJ, McKenzie JE, Bossuyt PM, Boutron I, Hoffmann TC, Mulrow CD (2021). The PRISMA 2020 statement: An updated guideline for reporting systematic reviews. Int. J. Surg..

[19] Alberti KGMM, Zimmet P, Shaw J (2006). Metabolic syndrome—A new world-wide definition. A consensus statement from the international diabetes federation. Diabet. Med..

[20] Sperling LS, Mechanick JI, Neeland IJ, Herrick CJ, Després J-P, Ndumele CE (2015). The cardiometabolic health alliance: Working toward a new care model for the metabolic syndrome. J. Am. Coll. Cardiol..

[21] Berhane T, Yami A, Alemseged F, Yemane T, Hamza L, Kassim M (2012). Prevalence of lipodystrophy and metabolic syndrome among HIV positive individuals on Highly Active Anti-Retroviral treatment in Jimma, South West Ethiopia. Pan Afr. Med. J..

[22] Tesfaye DY, Kinde S, Medhin G, Megerssa YC, Tadewos A, Tadesse E (2014). Burden of metabolic syndrome among HIV-infected patients in Southern Ethiopia. Diabetes Metab. Syndr. Clin. Res. Rev..

[23] Hirigo AT, Tesfaye DY (2016). Influences of gender in metabolic syndrome and its components among people living with HIV virus using antiretroviral treatment in Hawassa, southern Ethiopia. BMC Res. Notes.

[24] Bosho DD, Dube L, Mega TA, Adare DA, Tesfaye MG, Eshetie TC (2018). Prevalence and predictors of metabolic syndrome among people living with human immunodeficiency virus (PLWHIV). Diabetol. Metab. Syndr..

[25] Bune GT, Yalew AW, Kumie A (2020). The extents of metabolic syndrome among Antiretroviral Therapy exposed and ART naïve adult HIV patients in the Gedeo-zone, Southern-Ethiopia: A comparative cross-sectional study. Arch. Public Health.

[26] Ataro Z, Ashenafi W (2020). Metabolic syndrome and associated factors among adult HIV positive people on antiretroviral therapy in Jugal hospital, Harar, Eastern Ethiopia. East Afr. J. Health Biomed. Sci..

[27] Gebrie A (2020). The burden of metabolic syndrome in patients living with HIV/AIDS receiving care at referral hospitals of Northwest Ethiopia: A hospital-based cross-sectional study, 2019. Diabetes Metab. Syndr. Clin. Res. Rev..

[28] Bune GT, Yalew AW, Kumie A (2019). The global magnitude of metabolic syndrome among antiretroviral therapy (ART) exposed and ART-naïve adult HIV-infected patients in gedio-zone, southern Ethiopia: Comparative cross-sectional study, using the Adult Treatment Panel III criteria. Diabetes Metab. Syndr. Clin. Res. Rev..

[29] Sobieszczyk ME, Werner L, Mlisana K, Naicker N, Feinstein A, Gray CM (2016). Metabolic syndrome after HIV acquisition in South African women. JAIDS J. Acquir. Immune Defic. Syndr..

[30] Mashinya F, Alberts M, Van Geertruyden J-P, Colebunders R (2015). Assessment of cardiovascular risk factors in people with HIV infection treated with ART in rural South Africa: A cross sectional study. AIDS Res. Ther..

[31] Fourie CMT, Van Rooyen JM, Kruger A, Schutte AE (2010). Lipid abnormalities in a never-treated HIV-1 subtype C-infected African population. Lipids.

[32] Phalane E, Fourie CM, Schutte AE (2018). The metabolic syndrome and renal function in an African cohort infected with human immunodeficiency virus. South. Afr. J. HIV Med..

[33] Nguyen KA, Peer N, Mills EJ, Kengne AP (2016). A meta-analysis of the metabolic syndrome prevalence in the global HIV-infected population. PLoS One.

[34] Awotedu K, Ekpebegh C, Longo-Mbenza B, Iputo J (2010). Prevalence of metabolic syndrome assessed by IDF and NCEP ATP 111 criteria and determinants of insulin resistance among HIV patients in the Eastern Cape Province of South Africa. Diabetes Metab. Syndr. Clin. Res. Rev..

[35] Ojong E, Iya B, Djeufouata J, Ndeh F, Nsonwu A, Njongang V (2022). Metabolic syndrome and its components among HIV/AIDS patients on Antiretroviral Therapy and ART-Naïve Patients at the University of Calabar Teaching Hospital, Calabar, Nigeria. Afr. Health Sci..

[36] Muhammad S, Sani MU, Okeahialam BN (2013). Cardiovascular disease risk factors among HIV-infected Nigerians receiving highly active antiretroviral therapy. Niger. Med. J..

[37] Ayodele OE, Akinboro AO, Akinyemi SO, Adepeju AA, Akinremi OA, Alao CA (2012). Prevalence and clinical correlates of metabolic syndrome in Nigerians living with human immunodeficiency virus/acquired immunodeficiency syndrome. Metab. Syndr. Relat. Disord..

[38] Masyuko SJ, Page ST, Kinuthia J, Osoti AO, Polyak SJ, Otieno FC (2020). Metabolic syndrome and 10-year cardiovascular risk among HIV-positive and HIV-negative adults: A cross-sectional study. Medicine.

[39] Osoti A, Temu TM, Kirui N, Ngetich EK, Kamano JH, Page S (2018). Metabolic syndrome among antiretroviral therapy-naive versus experienced HIV-infected patients without preexisting cardiometabolic disorders in Western Kenya. AIDS Patient Care STDs.

[40] Chihota BV, Mandiriri A, Shamu T, Muula G, Nyamutowa H, Taderera C (2022). Metabolic syndrome among treatment-naïve people living with and without HIV in Zambia and Zimbabwe: A cross-sectional analysis. J. Int. AIDS Soc..

[41] Hamooya BM, Mulenga LB, Masenga SK, Fwemba I, Chirwa L, Siwingwa M (2021). Metabolic syndrome in Zambian adults with human immunodeficiency virus on antiretroviral therapy: Prevalence and associated factors. Medicine.

[42] Mbunkah HA, Meriki HD, Kukwah AT, Nfor O, Nkuo-Akenji T (2014). Prevalence of metabolic syndrome in human immunodeficiency virus-infected patients from the South-West region of Cameroon, using the adult treatment panel III criteria. Diabetol. Metab. Syndr..

[43] Muyanja D, Muzoora C, Muyingo A, Muyindike W, Siedner MJ (2016). High prevalence of metabolic syndrome and cardiovascular disease risk among people with HIV on stable ART in southwestern Uganda. AIDS Patient Care STDs.

[44] Tladi D, Mokgatlhe L, Nell T, Shaibu S, Mitchell R, Mokgothu C (2021). Prevalence of the metabolic syndrome among Batswana adults in urban and semi-urban Gaborone. Diabetes Metab. Syndr. Obes. Targets Ther..

[45] Zannou DM, Denoeud L, Lacombe K, Amoussou-Guenou D, Bashi J, Akakpo J (2009). Incidence of lipodystrophy and metabolic disorders in patients starting non-nucleoside reverse transcriptase inhibitors in Benin. Antivir. Ther..

[46] Bune GT, Yalew AW, Kumie A (2020). Predictors of metabolic syndrome among people living with HIV in Gedeo-Zone, Southern-Ethiopia: A case–control study. HIV/AIDS-Res. Palliat. Care.

[47] Guira O, Tiéno H, Diendéré AE, Sagna Y, Diallo I, Yaméogo B (2016). Features of metabolic syndrome and its associated factors during highly active antiretroviral therapy in Ouagadougou (Burkina Faso). J. Int. Assoc. Provid. AIDS Care (JIAPAC).

[48] Nguyen KA, Peer N, De Villiers A, Mukasa B, Matsha TE, Mills EJ (2017). Metabolic syndrome in people living with human immunodeficiency virus: An assessment of the prevalence and the agreement between diagnostic criteria. Int. J. Endocrinol..

[49] Woldu M, Minzi O, Engidawork E (2020). Prevalence of cardiometabolic syndrome in HIV-infected persons: A systematic review. J. Diabetes Metab. Disord..

[50] Nery MW, Martelli CMT, Turchi MD (2011). Dyslipidemia in AIDS patients on highly active antiretroviral therapy. Braz. J. Infect. Dis..

[51] Calza L, Colangeli V, Magistrelli E, Rossi N, Rosselli Del Turco E, Bussini L (2017). Prevalence of metabolic syndrome in HIV-infected patients naive to antiretroviral therapy or receiving a first-line treatment. HIV Clin. Trials.

[52] Lemieux I, Pascot A, Couillard C, Lamarche BT, Tchernof A, Alméras N (2000). Hypertriglyceridemic waist: A marker of the atherogenic metabolic triad (hyperinsulinemia; hyperapolipoprotein B; small, dense LDL) in men?. Circulation.

[53] Rütti S, Rohrer L, Donath M, von Eckardstein A (2009). S3–19 low and high density lipoproteins modulate function, apoptosis or proliferation of primary human and murine pancreatic beta cells. Atherosclerosis..

[54] Siebel AL, Natoli AK, Yap FY, Carey AL, Reddy-Luthmoodoo M, Sviridov D (2013). Effects of high-density lipoprotein elevation with cholesteryl ester transfer protein inhibition on insulin secretion. Circ. Res..

[55] von Eckardstein A, Sibler RA (2011). Possible contributions of lipoproteins and cholesterol to the pathogenesis of diabetes mellitus type 2. Curr. Opin. Lipidol..

[56] Onat A (2011). Metabolic syndrome: Nature, therapeutic solutions and options. Expert Opin. Pharmacother..

[57] Nduka CU, Stranges S, Sarki AM, Kimani PK, Uthman OA (2016). Evidence of increased blood pressure and hypertension risk among people living with HIV on antiretroviral therapy: A systematic review with meta-analysis. J. Hum. Hypertens..

[58] Martin-Iguacel R, Negredo E, Peck R, Friis-Møller N (2016). Hypertension is a key feature of the metabolic syndrome in subjects aging with HIV. Curr. Hypertens. Rep..

[59] Lundgren JD, Babiker AG, Gordin F, Emery S, Grund B, Sharma S (2015). Initiation of antiretroviral therapy in early asymptomatic HIV infection. N. Engl. J. Med..

